# Age and synovitis affect the results of the treatment of knee osteoarthritis with Microfragmented Autologous Fat Tissue

**DOI:** 10.1007/s00167-022-07139-4

**Published:** 2022-09-10

**Authors:** R. Ferracini, M. Alessio-Mazzola, B. Sonzogni, C. Stambazzi, C. Ursino, I. Roato, F. Mussano, A. Bistolfi, S. Furlan, L. Godio, D. Alotto, M. Formica

**Affiliations:** 1 Department of Surgical Sciences, University of Genova, Largo Rosanna Benzi 10, 16134 Genoa, Italy; 2 Ospedale Koelliker, Corso Galileo Ferraris 247/255, 10134 Turin, Italy; 3 IRCCS Ospedale San Raffaele, Orthopaedic and Trauma Unit, Via Olgettina 60, 20132 Milan, Milano Italy; 4 Department of Surgical Sciences, Bone and Dental Bioengineering Laboratory, CIR-Dental School, University of Turin, via Nizza 230, 10126 Turin, Italy; 5 Orthopaedic and Traumatology, Cardinal Massaia Hospital, Corso Dante 202, 14100 Asti, Italy; 6 First University Service, Pathologic Anatomy, Azienda Ospedaliero-Universitaria Città Della Salute, Corso Bramante 88, 10126 Turin, Italy; 7 Department of General Surgery and Special Surgery, Burns Center Unit, Unit of Skin Bank, Via Zuretti 29, 10126 Turin, Italy

**Keywords:** Knee osteoarthritis, Regenerative medicine, Adipose tissue-derived mesenchymal stem cells, Synovitis

## Abstract

**Purpose:**

This study aims to assess the effectiveness of Microfragmented Autologous Fat Tissue (MFAT) treatment for knee osteoarthritis and to investigate whether patients’ pre-treatment clinical condition, such as synovitis, correlates with clinical outcomes, to identify potential predicting factors for the success or failure of the treatment.

**Methods:**

In this prospective Cohort Study Level II multicentric trial, consecutive patients with a diagnosis of early/mild osteoarthritis and failure of previous conservative measures were enrolled to undergo diagnostic arthroscopy and a single MFAT injection. Patients were assessed with repeated scoring systems at baseline, 6 months, and 12 months after surgery. The demographic features, the arthroscopic findings, the immunophenotype of injected tissue and the histologic examination of synovia of failed patients were analyzed.

**Results:**

Data from 91 patients showed a significant improvement in Lysholm, WOMAC scores at 1-year follow-up (p < 0.001). A significant decrease in VAS score was observed, while a significant improvement of measured flexion angle was registered at 1 year (p < 0.001). No major complications were reported.

Age and synovitis were identified as significant factors influencing the clinical outcome (p < 0.05). Body mass index, previous or concomitant procedures, and specific cartilage defects had no influence. The mean number of injected adipose tissue-derived mesenchymal stem cells seem not to correlate with the clinical outcome.

**Conclusion:**

MFAT is effective in reducing pain when used with a single dose injection in early/mild OA of the knee, without major complications. Age over 60 and synovitis may be predictive for persistent pain at one year and should be considered before indications.

## Introduction

Knee osteoarthritis (OA) is an extremely common chronic disease worldwide affecting cartilage [10, 12, 13], thus cell-based cartilage tissue engineering approaches have been used to treat focal articular cartilage defects, with varying degrees of success [2, 5, 9, 15, 20, 23, 24]. Particularly studied is the use of mesenchymal stem cells (MSCs), derived from different tissues, which showed properties of self-renewal, multi-lineage differentiation, immune-modulation, and activation of the microenvironment by secreting different growth factors and cytokines [11, 19, 25, 27, 28]. Adipose tissue-derived mesenchymal stem cells (ASCs) are an attractive source for the treatment of joint damage due to the easiness of sourcing and handling, moreover, they are not affected by aging, maintaining the ability to differentiate in vitro into osteoblasts, chondrocytes, and adipocytes, according to the different stimuli received [8]. Nevertheless, the clinical use of adipose tissue and its derivatives, such as ASCs and a stromal vascular fraction (SVF) is still a field of debate in the orthopedic community, and it is strictly regulated by Food and Drugs Administration (FDA) and European Medical Agency (EMA) [30]. To overcome the problems concerning the expansion ex vivo of ASCs, different medical devices have been developed and approved for clinical use, allowing for minimally manipulate adipose tissue, which is then reinjected in the joint, demonstrating effectiveness [22]. This study analyzed a prospective series of consecutive patients affected by early/mild knee OA, treated with a single dose of Microfragmented Autologous Fat Tissue (MFAT), obtained through Lipogems® device [4], at a follow-up of 12 months. The purposes of the study were (i) to assess the effectiveness of MFAT treatment, (ii) to investigate whether patients’ pre-treatment clinical condition correlates with clinical outcomes, to identify potential predicting factors for success or failure of the treatment, and (iii) to evaluate whether the number of ASCs present in the injected MFAT correlates with the clinical outcome. The study of the biological characteristics of the implants compared to the clinical outcome, and the histological evaluation of the failed treatments represent an original approach, which allows to collection of novel information useful to improve MFAT treatment of OA patients.

## Material and methods

From June 2015 to December 2020, 101 consecutive patients, with a diagnosis of knee OA and failure of all previous conservative measures, were prospectively enrolled and were followed for 1 year. The patients underwent diagnostic arthroscopy and a single MFAT injection. The inclusion criteria were knee pain and functional impairment with a minimum of 1 year of symptoms, mild to moderate knee OA at standard X-ray examination (Kellgren Lawrence grade 2–3), presence of knee cartilage defects at 1.5 T magnetic resonance imaging (MRI) assessment, and failure of all previous conservative treatments (physiotherapy, cortisone, or hyaluronic acid and PRP intraarticular injections). Exclusion criteria were pregnancy or lactating, history of rheumatic diseases, coagulative disorders, history of alcohol or substance abuse, knee joint infections, skin disease or infections around the injection site, diabetes, previous articular knee fractures, severe medical illness based on the American Society of Anesthesiologists (ASA) score ≥ 3 [18]. All the patients had pre-operative knee standing antero-posterior and lateral X-ray view and 1.5 Tesla MRI investigations. The clinical protocol included a baseline clinical examination using Western Ontario and McMaster Universities Arthritis Index Score (WOMAC) and Lysholm Knee Score to assess functional disability. The visual analogue scale (VAS) was used to assess the subjective level of pain. Patients were prospectively assessed with repeated scoring systems at baseline, 6 months, and 12 months after surgery. Failure was defined as secondary knee replacement surgery, recurrence of symptoms within the 12 months following the procedure or poor Lysholm score at 1-year follow-up.

### Microfragmented Autologous Fat Tissue preparation and diagnostic arthroscopy

Harvest of adipose tissue was performed under spinal anesthesia in sterile conditions in the operating theatre. The patient was positioned supine, lower abdomen was the favorite donor site but in the case of low-fat mass, harvesting was performed from the lateral thighs subcutis. The adipose tissue was prepared for harvesting by injection of approximately 300 mL of Ringer solution containing 0.5 mg of adrenaline to reduce bleeding (as in modified Klein solution) [26].

Ten minutes after the infusion, approximately 80 mL of fat tissue were harvested manually using a 13G blunt suction cannula connected to a Vaclock® 20 ml syringe (Merit Medical Systems, Salt Lake City, UT, USA). At the end of the procedure, the skin was closed with steri-strips, and the patient was invited to wear a compression girdle for 3 weeks. MFAT tissue was prepared using the Lipogems® kit system (Lipogems International S.p.A., Milano, Italy) that mechanically reduces the size of the adipose tissue clusters while eliminating blood residues with pro-inflammatory properties during constant irrigation with saline solution. The obtained micro-fragmented tissue was collected in sterile 10 ml syringes for injection in the affected knee. MFAT injection was performed at the end of knee arthroscopy through one of the two arthroscopic portals under direct visualization. During the arthroscopy, intra-articular disorders of the knee including meniscal, synovial, ligamentous, and articular cartilage lesions were documented and treated when indicated. In the case of synovitis, partial synovectomy with werewolf coblation (ArthroCare Corporation, Austin, TX, USA) was performed.

Patients followed standardized post-operative protocols: partial weightbearing with two crutches for 2 weeks; quadriceps isometric exercises were recommended immediately after surgery and rehabilitation for a progressive range of motion exercises was conducted as tolerated.

### Immunophenotype analysis of Microfragmented Autologous Fat Tissue

MFAT samples were treated with collagenase IV (Serva GmbH) to retrieve SVF and analyze its various cell components, in particular the ASCs, expressing CD105 + , CD73 + , CD90 + and CD44 + and negative for HLA-DR, CD34, CD31 and CD45; CD146 + pericytes, CD31 + endothelial cells, CD34 + hematopoietic cells. The standard labelling protocol was performed with the following fluorochrome-conjugated antibodies and isotypic controls: ASCs were marked with MSC phenotyping kit, human CD44 PE, CD45 PerCP, IgG1 PE, IgG1 FITC, IgG1 APC, and IgG2a PerCP (Miltenyi Biotec, Bergisch Gladbach, Germany), CD31PE, CD34FITC, CD146APC and CD90 PerCP (Biolegend, San Diego, CA, USA). About 10^5^ events/sample were used for capture with MACsQUANT10 and data were analyzed with MACs quantify software (Miltenyi Biotec, Bergisch Gladbach, Germany). The percentage of ASCs in the MFAT volume was determined, and then the number of ASCs for ml of MFAT was normalized on the absolute number of viable cells.

### Histological examination

The tissues retrieved from patients who underwent knee arthroplasty for failure were treated in the pathology department, according to the standard protocols to obtain paraffin-embedded tissues. These tissues have been matched based on demographic features and compared with those from OA patients operated for total knee arthroplasty not previously injected with MFAT. Hematoxylin and eosin (H&E) preparation and optical microscopy techniques have been used for analyzing the tissue types and the morphologic changes. Sections of synovial membranes were obtained for morphological analysis.

### Statistical analysis

A power analysis was conducted to calculate the sample size. For a statistical power of 80% and a level of significance of 0.01, a total of 86 cases are needed to observe a significant difference between the baseline and the final values of Lysholm score with an expected increase of 5 points. Considering a possible dropout of 15%, 101 patients were prospectively enrolled. The Shapiro–Wilk Test was used to calculate the normal distribution of variables. Categorical variables were expressed as the absolute number of cases and percentage. Continuous variables of independent groups were compared with Mann–Whitney *U* test. The repeated measures analysis of variance (ANOVA) with Post Hoc test was used to compare continuous outcome values at different timepoints. Categorical variables were compared using the Chi-square test or Fisher’s exact test. The Pearson coefficient was used to assess the correlation between continuous variables. Variables achieving the *p* value < 0.1 in univariate analysis were examined using multivariate linear regression analysis. For all variables, statistical significance was set at *p* < 0.05.

## Results

Ninety-one patients concluded the study, while 10 patients were lost to follow-up with a final drop-out of 9.9%. All patients received a single dose of intra-articular injection of MFAT after knee arthroscopy. Table 1 resumes the baseline features of the study population and the type of accessory arthroscopic procedures.

**Table 1:** Demographics and general features of overall population (*N* = 91) who underwent arthroscopic MFAT injection

Number of patients	91
Age at surgery (years)	62.8 ± 10.1 (36–84)
Gender (female/male)	47 (51.6%)/44 (48.4%)
Side (right/left)	51 (56.0%)/40 (44%)
Injection volume (mL)	26.8 ± 10.1 (3 to 50)
BMI	25.3 ± 3.8 (20.0–37.1)
Combined procedures	33 (36.3%)
Medial meniscectomy	26 (28.6%)
Lateral meniscectomy	3 (3.3%)
ACL reconstruction	4 (4.4%)
Previous surgeries (yes/no)	4 (5.3%)/87 (94.7%)

Continuous variables are expressed with main values, standard deviation, and range of values (under parenthesis)

Absolute values are expressed by frequencies and relative percentage (under parenthesis)

**Fig. 1:**
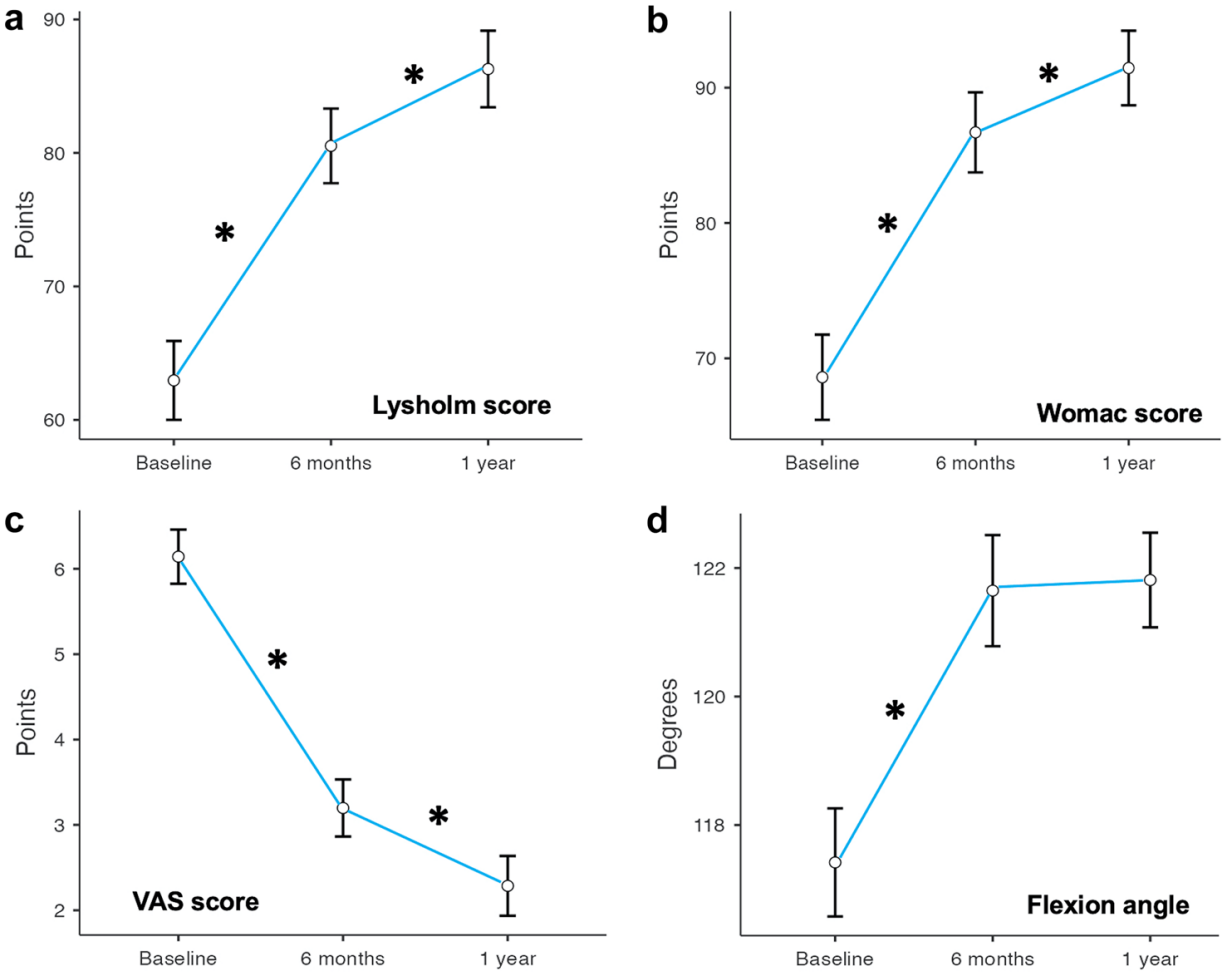
Graphical representation of clinical outcome values. **A** Lysholm score, **B** Womac score, **C** visual analog scale (VAS), **D** Flexion angle. Asterisks highlight significant differences between measurements (repeated measure ANOVA with post hoc test)

**Fig. 2:**
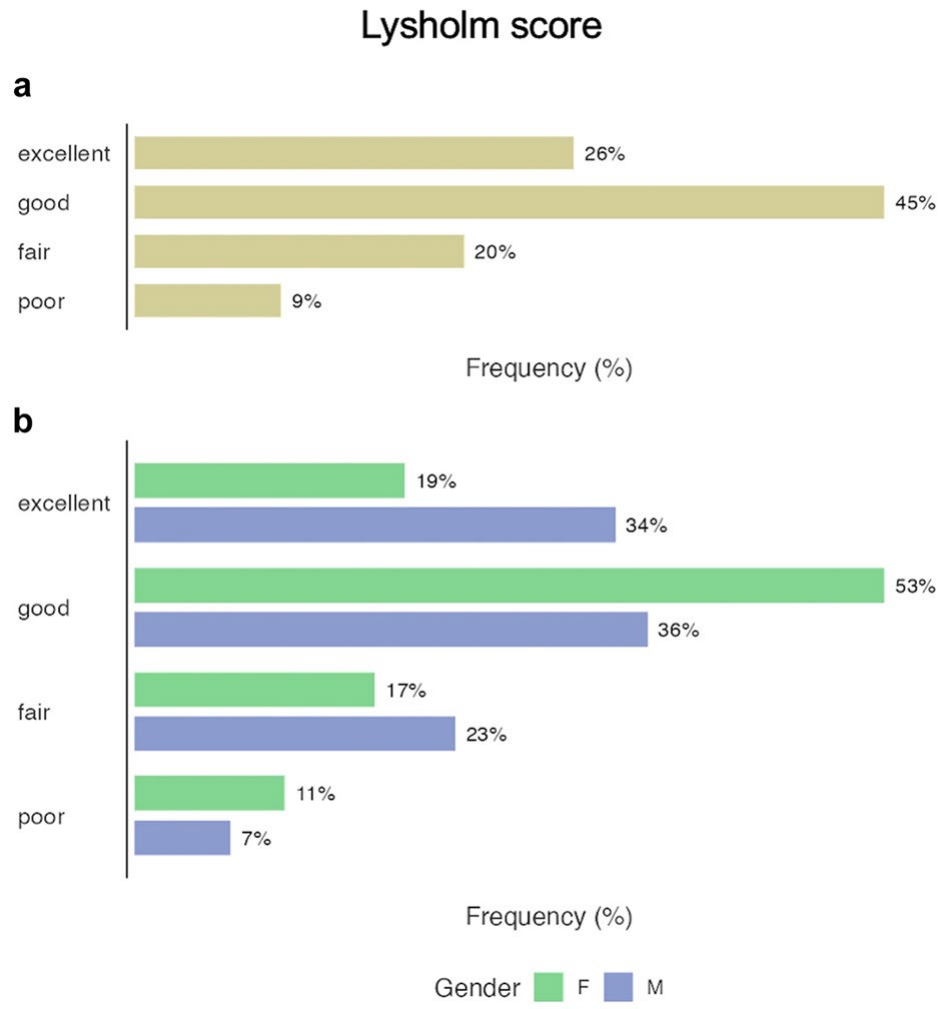
Graphical expression of clinical outcome results of Lysholm score at 1 year. **A** overall results, **B** results stratified for gender

At 1 year follow-up, Lysholm and WOMAC scores significantly improved as well as the measured flexion angle, while VAS score decreased (detailed are reported in Table 2 and Fig. 1). At 1 year, the failure rate was 8.8% without significant differences among men and women in all assessed clinical outcomes (Fig. 2). Patients who failed showed significantly higher mean age at surgery, moreover, among these patients, 5 had intra-articular synovitis detected at diagnostic arthroscopy.

**Table 2:** Prospective comparisons of clinical outcome values at different timepoints of follow-up with mean values, standard deviations (SD), range of values, 95% confidence intervals (95% CI), mean differences, standard errors (SE) and *p* values

Lysholm	Baseline	6 months	1 year
Mean ± SD	63.0 ± 14.2	80.5 ± 13.4	86.3 ±13.8
Range	40.0–84.0	37.2–100.0	40.0–100.0
95% CI	60.0–65.9	77.7–83.3	83.4–89.2

		Mean Difference	SE	*p* value
Baseline	6 months	− 17.57	1.883	< .001 *
6 months	1 year	− 23.33	1.958	< .001 *
	1 year	− 5.76	0.216	< .001 *

Womac	Baseline	6 months	1 year
Mean ± SD	68.6 ± 15.1	86.7 ± 14.2	91.5 ± 13.2
Range	43.5–86.0	40.4–100.0	43.5–100.0
95% CI	65.5–71.7	83.7–89.7	88.7–94.2

		Mean Difference	SE	*p* value
Baseline	6 months	− 18.10	2.035	< .001 *
6 months	1 year	− 22.86	2.014	< .001 *
	1 year	− 4.76	0.371	< .001 *

VAS	Baseline	6 months	1 year
Mean ± SD	6.1 ± 1.5	3.2 ± 1.6	2.3 ± 1.7
Range	1.0–8.0	1.0–8.0	1.0–8.0
95% CI	5.8–6.5	2.9–3.5	1.9–2.6

		Mean Difference	SE	*p* value
Baseline	6 months	2.945	0.197	< .001*
6 months	1 year	3.857	0.222	< .001*
	1 year	0.912	0.106	< .001*

Flexion angle	Baseline	6 months	1 year
Mean (SD)	117.4 ± 4.0	121.6 ± 4.2	121.8 ± 3.5
Range	100.0–125.0	100.0–125.0	105.0–125.0
95% CI	117.0–118.0	121.0–123.0	121.0–123.0

		Mean Difference	SE	*p* value
Baseline	6 months	− 4.231	0.220	< .001*
6 months	1 year	− 4.396	0.280	< .001*
	1 year	− 0.165	0.182	0.369

Data were compared with repeated measured analysis of variance (ANOVA) and post hoc test. Asterisks highlight significant data

### Correlation between the clinical factors and the outcomes

There was a significant negative correlation among age at surgery, Lysholm and WOMAC scores at 6 months and 1 year, reflecting that patients with lower age at surgery demonstrated higher clinical scores at 6 months and 1 year, Table 3.

**Table 3:** Comparisons of clinical outcome values of patients stratified for age with the relative number of subgroups expressed by means ± standard deviations and range of values (under parenthesis)

	< 60 years	≥ 60 years	*p* value
	(*n* = 41)	(*n* = 50)	
Baseline			
Lysholm	63.9 ± 15.8 (40–84)	61.9 ± 13.2 (40–83)	0.461
Womac	69.6 ± 16.8 (43.5–84)	67.5 ± 14.1 (43.5–86)	0.426
VAS	6.0 ± 1.5 (3–8)	6.2 ±1.6 (3–8)	0.626
Flexion angle	118.1 ± 2.7 (110–120)	116.7 ± 4.9 (100–125)	0.217
6 months			
Lysholm	85.5 ± 10.6 (45.6–100)	82.9 ± 15.7 (40.4–100)	0.004*
Womac	91.6 ± 10.7 (49.5–100)	3.5 ± 1.5 (1–7)	0.006*
VAS	2.7 ± 1.6 (1–8)	76.7 ± 14.5 (37.2–93)	<0.001*
Flexion angle	122.4 ± 2.5 (120–125)	120.8 ± 5.0 (100–125)	0.174
1 year			
Lysholm	91.0 ± 10.6 (49–100)	82.7 ± 15.2 (40–100)	0.002*
Womac	95.6 ± 9.6 (53.3–100)	88.2 ± 15.0 (43.5–100)	0.003*
VAS	1.9 ± 1.7 (1–8)	2.5 ± 1.5 (1–7)	0.008*
Flexion angle	122.4 ± 2.5 (120–125)	121.1 ± 4.1 (105–125)	0.174

Asterisks highlight significant data

Multivariate analysis showed that synovitis and age at surgery were both significant independent variables related to poor Lysholm score at 6 months (Adjusted *R*2 = 0.146; *p* < 0.001) and 1 year (Adjusted *R*2 = 0.141; *p* < 0.001) and Womac score at 6 months (Adjusted *R*2 = 0.147; *p* < 0.001) and 1 year (Adjusted *R*2 = 0.122; *p* = 0.001

Both after 6 and 12 months, there was a positive correlation between age and VAS score, therefore patients with higher age at surgery registered a higher level of pain. Patients with age at surgery ≥ 60 years had a significantly lower clinical outcome. No significant correlations were found between age and pre-operative values, Table 3.

Patients with arthroscopic evidence of synovitis demonstrated significantly lower values of Lysholm score at 6 months and 1 year. WOMAC scores were lower at 6 months and significantly decreased at 1 year.

No differences were found between flexion angle and VAS scores among patients with synovitis at all endpoints, Table 3.

No correlations between BMI and outcome variables were detected and there were no differences in clinical outcome values among patients stratified for BMI ≥ 25 points and for BMI ≥ 30 at all timepoints.

Previous knee surgery or concomitant arthroscopic partial meniscectomy (medial or lateral) had no influence on clinical outcomes values. There were no differences in clinical outcome values at all timepoints among subgroup of patients who underwent combined procedures (*n* = 33) or isolated arthroscopic MFAT injection (*n* = 58).

**Fig. 3:**
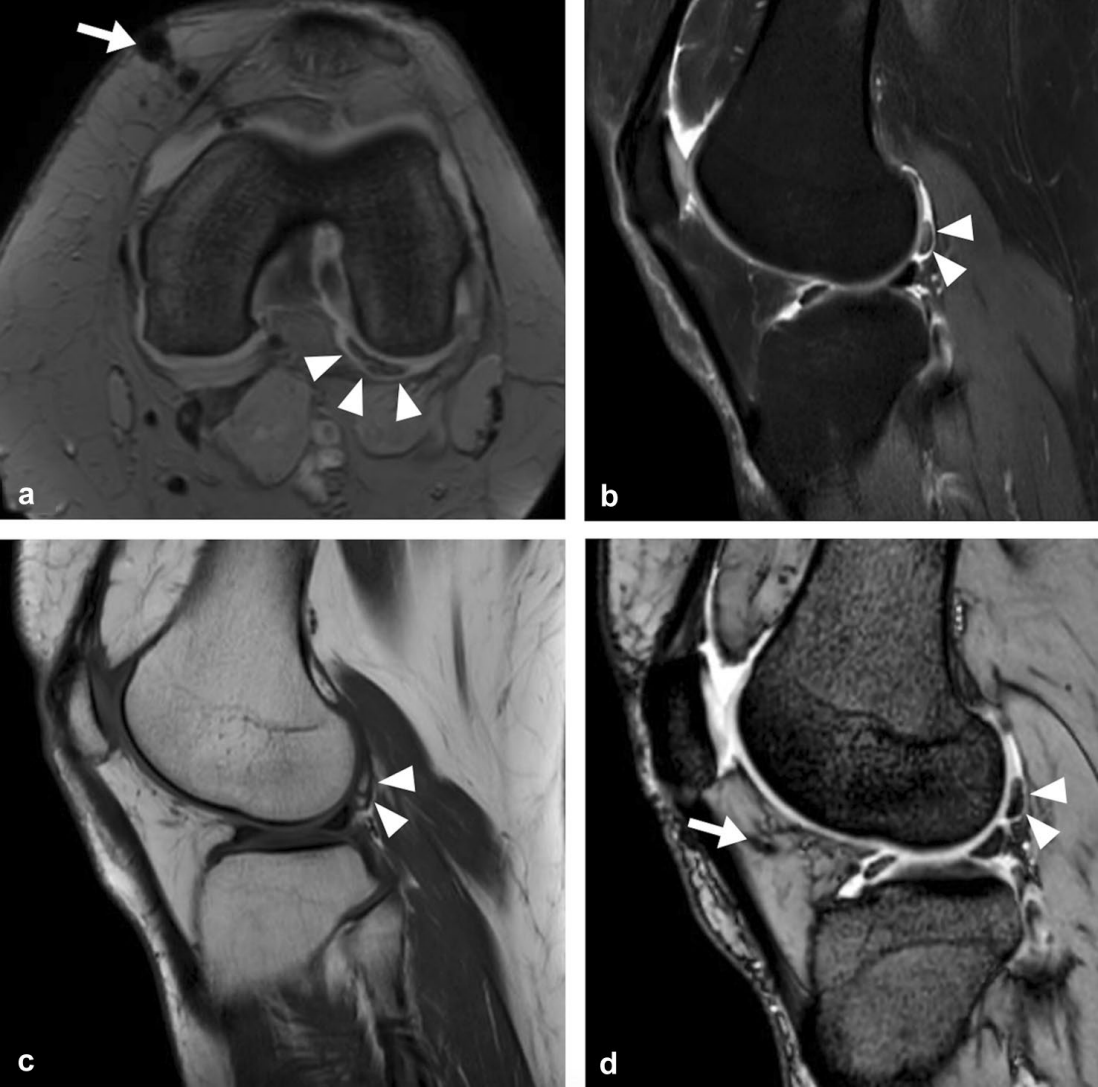
**A** T2-weighted sm-FFE axial image (Achieva 1.5 T Philips MRI scanner, with dStream technology). A large loose adipocyte intraarticular body in the posterolateral corner circumferentially surrounded by fluid effusion. The intraarticular body is hypointense, well defined (white arrowheads). On the medial anterior side the magnetic susceptibility artifact due to surgical scar (white arrow). **B**-**D** sagittal plane (Achieva 1.5 T Philips MRI scanner, with dStream technology): fat sat sequence sm-FFE and mFFE fat sat, T1 weighted TSE confirm adipose content of intraarticular loose bodies, with regular margin and homogenous internal structure that is hypointense with fat saturation and hyperintense in T1 -weighted FSE sequence (white arrowheads). On the anterior side the magnetic susceptibility artifact due to surgical scar (white arrow)

*Complications:* No major complications occurred during follow-up. No complications were described at the site of fat tissue harvesting. Swelling, minor inflammatory infiltration and subcutaneous hematoma were fading in 3 weeks on average and were not considered complications. Four (4.4%) patients had minor articular complications within the study period: 2 patients developed painful adipose loose bodies 1 month after surgery and required additional 1.5 Tesla MRI investigation (Fig. 3). The symptoms spontaneously resolved within 3 months and the patients improved at 6 months and 1-year follow-up. One patient had recurrent episodes of joint effusions during the first 6 months after surgery, nevertheless, the clinical scores detected significant clinical improvement at 6 months and 1 year, respectively, and the patient did not require additional treatment.

### Immunophenotype results of MFAT

**Fig. 4:**
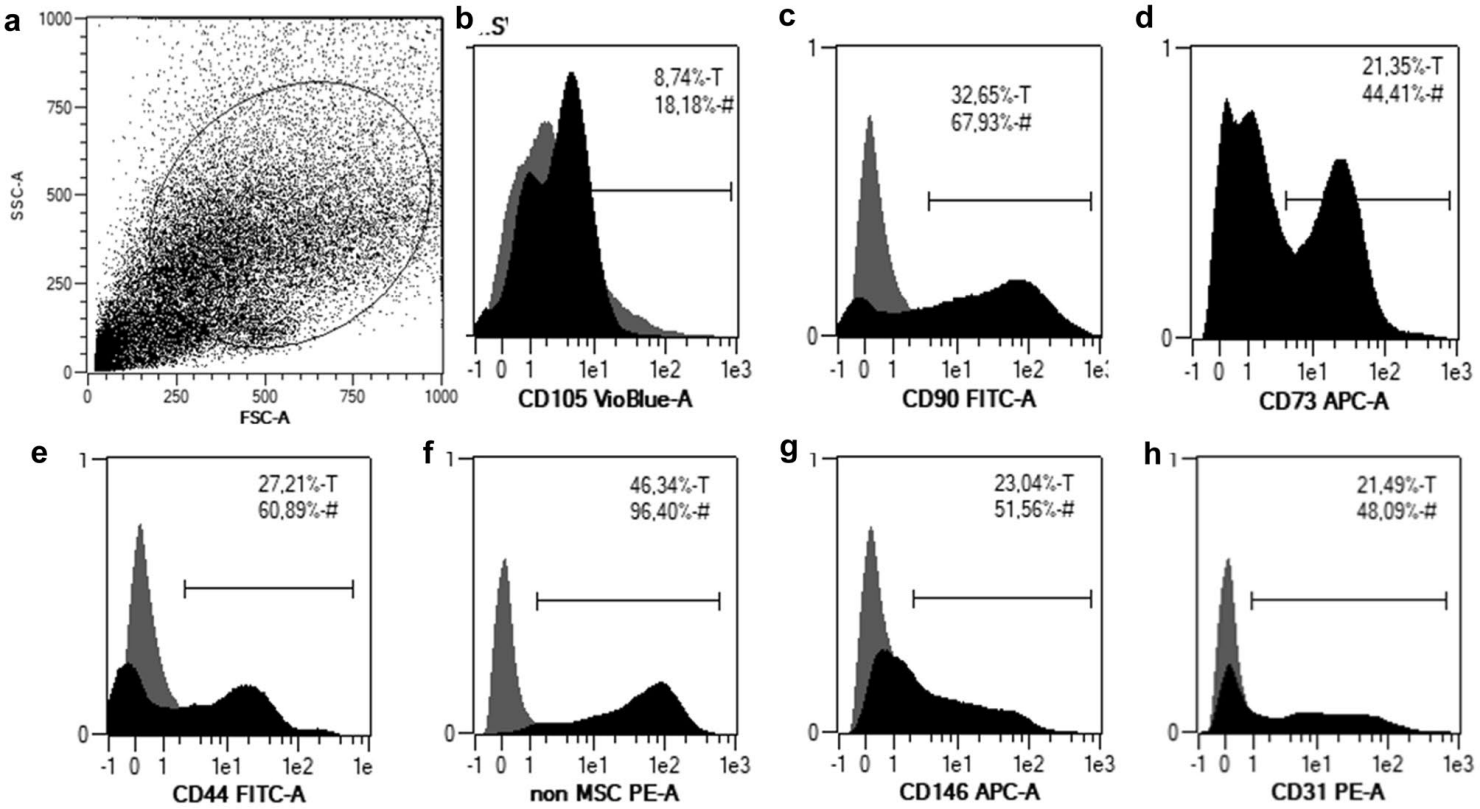
Micro-fragmented fat contains derived stem cells which are present in stromal vascular fraction. SVF is a heterogeneous population of cells (**A**), containing also ASCs, characterized by the expression of different markers (**B**-**E**). # and T indicate the % of positive cells in the gate, and on the total of the sample, respectively. The presence of adipose-derived stromal cells is identified by the expression of typical markers, such as CD105, CD90, CD73, CD44 (Fig. 4**B**-**E**), but there are also non- mesenchymal cells (CD34, CD45, CD14, CD19, HLA-DR negative cells) (Fig. 4**F**), perycites (Fig. 4**G**) and endothelial cells (Fig. 4**H**)

MFAT contains the stromal vascular fraction (SVF), which is constituted by a large variety of cell subpopulations, as revealed by the immunophenotype analysis (Fig. 4) The presence of adipose-derived stromal cells is identified by the expression of typical markers, such as CD105, CD90, CD73, CD44, but there are also non-mesenchymal cells (CD34, CD45, CD14, CD19 positive and HLA-DR negative cells), pericytes and endothelial cells. The mean number of ASCs expressing CD105 + , CD73 + , CD90 + and negative for CD45 in injected MFAT was 0.974 *10^6^ ± 0.059 * 10^6^ cells (range: 0.005 * 10^6^ to 0.168 * 10^6^). No significant correlation was found either between the number of retrieved and injected mesenchymal stem cells with patients’ age at surgery (*p* = 0.869) or with clinical outcome values at 6 months (Lysholm: *p* = 0.193; WOMAC: *p* = 0.787) and 1 year (Lysholm: *p* = 0.297; WOMAC: *p* = 0.398).

### Histological results

**Fig. 5:**
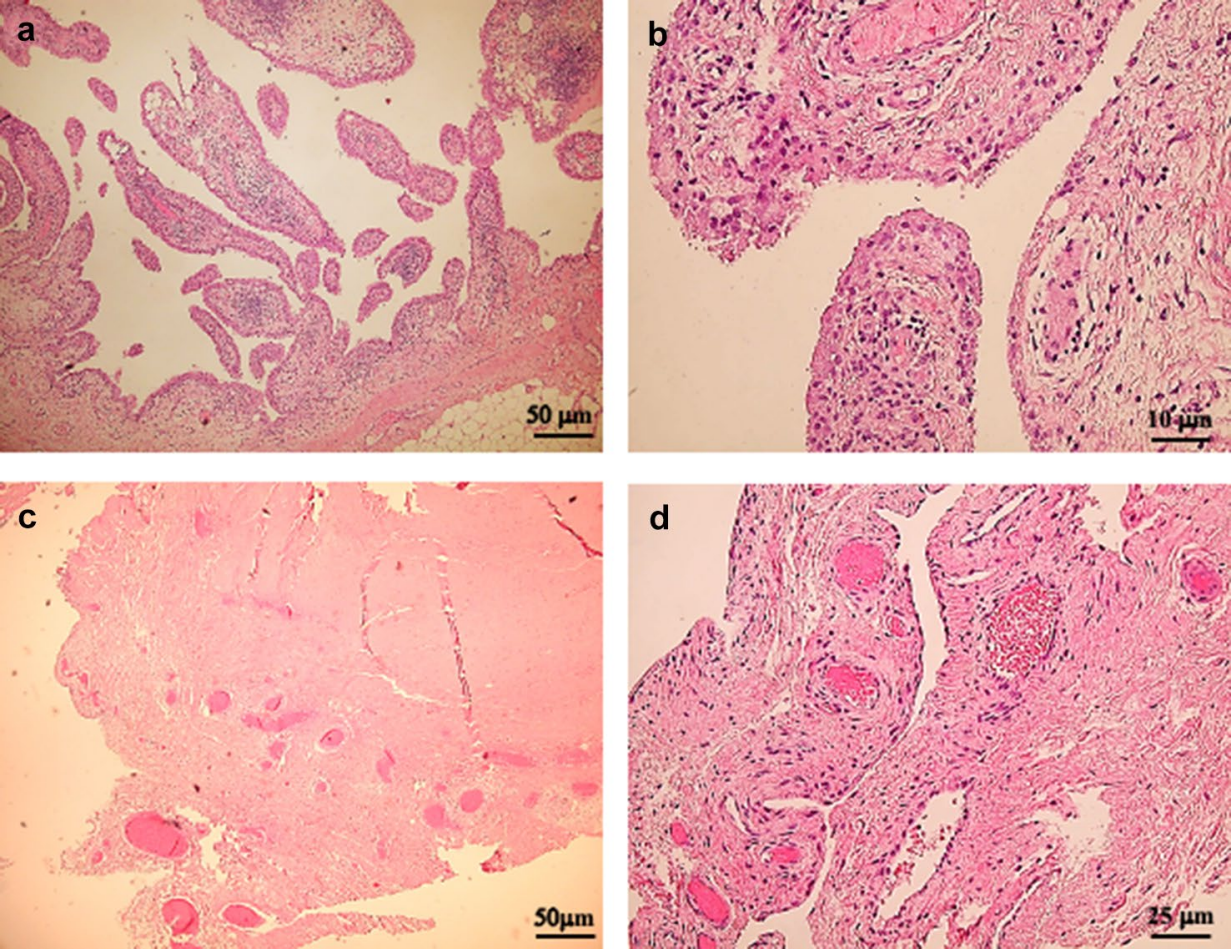
Hematoxylin—Eosin (HE) staining of the synovial membrane of OA patients not treated with MFAT compared with that of treated patients. The histological analysis of synovial membranes from OA patients, who were or were not subjected to MFAT treatment before arthroplasty, showed marked hyperplasia, characterized by the presence of villous structures, multilayered chondrocytes, and a massive infiltrate of lymphocytes in untreated patients, Fig. 5**A**, **B**, while an inferior level of hyperplasia and lymphocyte infiltrate was present in treated patients, Fig. 5**C**, **D**

The histological results showed that the MFAT treatment positively affects the state of the synovial membrane. Among the failed MFAT-treated patients, 4 underwent knee arthroplasty at an average of 24 months follow-up. A comparative histological analysis of synovial membranes from OA patients who were or were not subjected to MFAT treatment before arthroplasty showed different histological findings: a marked hyperplasia, characterized by the presence of villous structures, multilayered chondrocytes, and a massive infiltrate of lymphocytes in untreated patients, while an inferior level of hyperplasia and lymphocyte infiltrate was present in treated patients, Fig. 5.

## Discussion

This study showed a clinically meaningful improvement in knee OA symptoms and pain at 6 months and 1 year after intra-articular injection of MFAT extracts. In addition, it identified two important factors which may negatively affect the results: age and synovitis. On the other hand, BMI, sex and isolated chondral lesions did not affect the clinical results. In selected cases, we described intra-articular transitory hypertrophy of the adipose tissue, which resolved without treatment or further complications.

The literature reported several clinical trials showing a general improvement of the clinical symptoms [8, 15, 30]. A previous study from our group concluded that adipose tissue infusion stimulates tissue regeneration and might be considered a safe treatment for knee OA [26]. A recent work showed that patients treated at earlier-degeneration stages reported statistically significant improvements in VAS, WOMAC and walking distance [22]. Similarly, several recent systematic reviews and meta-analysis reported favorably on the use of MSCs [1]. Wiggers et al. found a positive effect of autologous MSC therapy compared with control treatments on patient-reported outcome measures, and disease severity [29]. Meng’s revision article documented a significant improvement in WOMAC, VAS, KOOS and an end point MOCART scores, but strongly suggested to establish standardized methods for MSC extraction and delivery, and to perform studies with longer follow-up [21].

Several potential factors influencing the clinical outcomes have been systematically assessed with better clinical results in patients younger than 60 without active inflammatory joint processes.

Surprisingly, variables which may have thought to have a potential impact on the results turned out with no clinical impact: BMI, sex, previous surgeries, and accessory surgery had no influence on clinical outcomes values. The associated arthroscopic surgical procedure may be questionable; nevertheless, we believe that it is necessary to remove potential sources of pain such as meniscal pathologies, loose bodies, etc. and, moreover, to eliminate the excess synovial fluid which is rich in inflammatory elements and may be a cause of persistent pain [3].

On the contrary, age is a parameter that should be considered for indications: according to our results, patients with lower age at surgery had higher clinical scores, while patients with higher age had a higher pain level. Definitely, age at surgery over 60 years may correlate with negative results. Another impacting aspect is the presence of arthroscopic evidence of synovitis; indeed, this group of patients demonstrated significantly lower WOMAC and Lysholm scores at the final follow-up. The presence of synovitis has no apparent impact on the range of movement and pain, but it has a clinical impact on the recovery of function, likely due to the highly inflamed synovial tissue, which might negatively interfere with the nesting of the mesenchymal progenitor cells. Indeed, it is known that low-grade synovial inflammation is a key aspect of the evolution of OA [17]. Clinical results are supported by *in-vitro* study by Filardo et al. [6] showing homeostatic changes of synovial tissue in MFAT treated samples due to the proprieties of ASCs to reduce typical macrophages markers, inducing specific inhibitors of synoviocytes. This therapeutic effect may be hindered whether active inflammatory processes are present at the time of surgery, explaining poor clinical outcomes of patients with arthroscopic evidence of intra-articular synovitis. The histologic analysis of the synovias from patients treated with MFAT and retrieved during further knee arthroplasty showed a reduced inflammatory condition in the synovial tissue compared to a cohort of non-MFAT pre-treated patients, who underwent the same arthroplasty procedure for OA. These data are in accordance with the known anti-inflammatory activity of ASCs [7, 14]. Therefore, our results suggest that the presence and the quality of synovitis should be considered pre-operatively and eventually treated. We believe that a specific MRI or ultrasound scan can be sufficient and appropriate to identify synovitis.

According to the immunophenotype of MFAT, the SVF is highly represented in all its cell subpopulations, among them ASCs showed large variability in the number of cells retrieved from different patients. Nonetheless, ASC number does not correlate with the clinical outcome of the treatment according to our previously published data [4]. Furthermore, a recent meta-analysis reporting data from different clinical trials, all with autologous MSC-based treatment of knee OA, the range of doses used was extremely variable and there was no clear evidence of a dose-response effect [16].

In this series, adipose tissue infusion was not associated with any adverse event such as chondro-toxicity, allergies, or implant rejection. Several patients had mild knee swelling associated with pain during the first weeks after surgery and one patient (1.1%) had recurrent episodes of joint effusions during the first 6 months after surgery. In all cases, clinical scores improved at the final follow-up. These reactions can be partly due to the volume of material infused; indeed, we prefer to harvest as much as possible adipose tissue (approximately 200 cc and no less than 100 cc) to inject into the joint the largest amount of ASCs (the final volume is around 20 ml of concentrate from adipose extract).

This study has some inherent limitations: it is a prospective study with a relatively short follow-up, the number of patients is limited, patients underwent different surgical treatments, and a direct or indirect description of the cartilage repair was not performed. Nevertheless, the number of patients has been estimated through a power analysis. The final follow-up at 1 year was aimed at the design of the study since the Authors believe it is a sufficient time to document the incidence of major complications and to evaluate the clinical outcome of the treated knees.

Finally, a quantification of the thickness, consistency and status of the cartilage is not feasible in this kind of study, because the MRI is not sufficient, and a biopsy and the consequent histological evaluation are not applicable in all cases for ethical reason.

## Conclusions

Microfragmented Autologous Fat Tissue is effective in reducing pain when used with a single dose injection in early/mild OA of the knee, without major complications. Age over 60 and synovitis may be predictive for persistent pain at one year and should be considered before indications.
